# ERK Phosphorylation Regulates the Aml1/Runx1 Splice Variants and the TRP Channels Expression during the Differentiation of Glioma Stem Cell Lines

**DOI:** 10.3390/cells10082052

**Published:** 2021-08-10

**Authors:** Giorgio Santoni, Massimo Nabissi, Consuelo Amantini, Matteo Santoni, Lucia Ricci-Vitiani, Roberto Pallini, Federica Maggi, Maria Beatrice Morelli

**Affiliations:** 1 Immunopathology Laboratory, School of Pharmacy, University of Camerino, 62032 Camerino, Italy; massimo.nabissi@unicam.it (M.N.); federica.maggi@uniroma1.it (F.M.); 2 Immunopathology Laboratory, School of Biosciences and Veterinary Medicine, University of Camerino, 62032 Camerino, Italy; consuelo.amantini@unicam.it; 3 Medical Oncology Unit, Hospital of Macerata, 62100 Macerata, Italy; mattymo@alice.it; 4 Department of Hematology, Oncology and Molecular Medicine, Istituto Superiore di Sanità, 00161 Rome, Italy; lucia.riccivitiani@iss.it; 5 Institute of Neurosurgery, Gemelli University Polyclinic Foundation, Scientific Hospitalization and Care Institute (IRCCS), 00168 Rome, Italy; roberto.pallini@policlinicogemelli.it; 6 Institute of Neurosurgery, School of Medicine, Catholic University, 00168 Rome, Italy; 7 Department of Molecular Medicine, Sapienza University, 00161 Rome, Italy

**Keywords:** glioma stem cells, neural differentiation, Aml1 splice variants, ERK, TRP channel, TRPV1, TRPA1

## Abstract

The identification of cancer stem cells in brain tumors paved the way for new therapeutic approaches. Recently, a role for the transcriptional factor Runx1/Aml1 and the downstream ion channel genes in brain cancer development and progression has been suggested. This study aimed to explore the expression and the role of Runx1/Aml1, its Aml1b and Aml1c splice variants and the downstream TRPA1 and TRPV1 ion channels in undifferentiated and day-14 differentiated neural stem cells (NSCs and D-NSCs) and glioblastoma stem cells (GSCs and D-GSCs) lines with different proneural (PN) or mesenchymal (MES) phenotype. Gene and protein expression were evaluated by qRT-PCR, cytofluorimetric, western blot and confocal microscopy analyses. Moreover, by western blot, we observed that ERK phosphorylation enhances the Aml1b and Aml1c protein expression during glioma differentiation. Furthermore, the agonists of TRPA1 and TRPV1 channels stimulated apoptosis/necrosis in GSCs and D-GSCs as evaluated by Annexin V and PI staining and cytofluorimetric analysis. Finally, by qRT-PCR, the modulation of Wnt/β catenin, FGF, and TGFβ/SMAD signaling pathways in PN- and MES-GSCs was reported. Overall, our results provide new evidence regarding Runx1/Aml1 isoform overexpression and modulation in TRP channel expression during gliomagenesis, thus offering new directions for glioblastoma therapy.

## 1. Introduction

Malignant gliomas remain one of the more deadly human brain tumors with a poor prognosis despite years of research in anti-tumor therapeutic strategies. A hallmark characteristic of gliomas is their molecular and cellular heterogeneity, which is considered the reason for their high malignancy, recurrence and resistance to therapy [1]. Neoplastic transformation of differentiated glial cells was the most accepted hypothesis to explain the origin of gliomas for many years [1,2]. However, recent reports support the existence of a stem cell-derived origin for different types of cancers, including brain tumors [3].

Neural stem cells (NSCs) and glial progenitor cells are present in multiple regions of the adult brain (e.g., the dentate gyrus, within the hippocampus and the subcortical white matter) [2]. The largest of these germinal regions in humans, the sub-ventricular zone, contains a population of astrocytes that can function as NSCs. In other adult mammals, glial progenitor cells, self-renewing precursors capable of producing NSCs, which are multipotent and self-renewing, have been isolated from the sub-ventricular zone. These stem cells and progenitor elements, in addition to differentiated adult glia, constitute a substrate for neoplastic transformation [3].

In regard to GSCs, they have been isolated from both human tumor tissues [4,5,6,7,8] and glioma cell lines [6,9,10,11]. In the same manner of NSCs, the GSCs retain the ability to respond to physiological signals that induce NSCs differentiation into neurons, astrocytes and oligodendrocytes [12]. The GSCs isolated from human brain tumors undergo self-renewal and multilineage cell differentiation [11,13]. Previous studies unveiled the inter- and intra-tumor heterogeneity of GSCs. At transcription levels, at least two mutually distinct subtypes of GSCs, a proneural-like (PN-like) and a mesenchymal-like (MES-like) phenotype, were identified [14,15]. MES-like GSCs showed higher rates of proliferation in vitro and in vivo; they are more angiogenic, invasive and resistant to radiation than PN-like GSCs [16]. Moreover, it was found that primary treated PN-like GBM often relapse, as tumors having MES-like markers become resistant to therapy [15,17].

The transcriptional network regulating GSCs proliferation and differentiation is only partially identified, indicating some drivers of tumor initiation, such as the Runt-related transcription factor 1 (Runx1), named Acute myeloid leukemia 1 (Aml1) in human [18,19]. The *Aml1* gene generates at least three alternatively spliced variants: *Aml1a*, *b* and *c*. Aml1b and Aml1c possess the DNA-binding region and the transcriptional regulatory domains and they are considered similar in functions. Aml1a retains the DNA-binding domain, but lacks the transcriptional regulatory domains and it is considered a potential functional antagonist of Aml1b and Aml1c [20].

In normal human brain, Aml1/Runx1 plays a relevant role in sensory neuron differentiation, controlling the expression of several sensory nociceptive receptors, including members of the transient receptor potential (TRP) class of thermal and mechano-receptors (e.g., TRPV1, TRPV2, TRPM8, TRPA1 and TRPC3) [21,22]. In human cancers, the *Runx1* gene has been reported to be mutated, as somatic point mutations in myelodysplasia and chromosomal translocation in acute myeloid leukemia [23]. Aberrant expression of *Aml1* in breast cancer [24] and esophagus adenocarcinoma [25] has been documented; moreover, *Aml1* is overexpressed in endometrial carcinoma [26] and downregulated in gastric cancer [27]. It was also found expressed in human glioma cell lines [18]. In this regard, *Aml1a* is upregulated during GSCs differentiation, and its downregulation restores a stem cell phenotype in differentiated GSCs [28]. 

The malignant transformation of cells resulting from enhanced proliferation and aberrant differentiation is often accompanied by changes in TRP channel expression and consequently by abnormal progression of the cellular responses. In this context, we have previously reported that the TRPV2 channel is involved in GSCs differentiation by interacting with Aml1. The Aml1a isoform binds the TRPV2 promoter and its expression is upregulated by TRPV2 activation in a PI3K/AKT-dependent manner. Additionally, the TRPV2 agonist, cannabidiol (CBD), by inducing a TRPV2-dependent autophagic process, stimulates Aml1a-dependent GSCs differentiation, abrogating the BCNU chemoresistance [28].

Despite the importance of Aml1/Runx1 transcriptional factor in hematological cancers [23], the regulatory mechanisms involved in the controlling *Aml1/Runx1* gene transcription during gliomagenesis remain poorly understood. Instead, the identification of the transcriptional factors responsible for altered differentiation in different GSCs subtypes and the distinct molecular signaling pathways could be important to organize new therapeutic strategies.

The aim of this work was to evaluate the expression of the alternative splice variants of the Runx1/Aml1 transcriptional factor (e.g., Aml1b and Aml1c) and to investigate how they are modulated during GSCs differentiation. In addition, the signaling pathway and the downstream TRP ion channels (e.g., TRPV1 and TRPA1) in PN- and MES-like GSCs was evaluated.

## 2. Materials and Methods

### 2.1. Cell Cultures

NSCs (NSC#1, NSC#2), proneural-like (PN-GSC#1, #23C, #28, #68 and #70) and mesenchymal-like (MES-GSC#30 and #83) GSC lines, previously characterized in [14], were isolated from surgical samples of seven adult patients with primitive brain tumor undergoing complete or partial surgical resection at the Institute of Neurosurgery, Catholic University School of Medicine, in Rome, Italy. Patients were eligible for the study if a diagnosis of glioblastoma multiforme was established histologically according to the WHO classification [29]. Informed consent was obtained before surgery according to the Ethical Committee of Catholic University School of Medicine. GSC cultures were established from tumor specimens through mechanical dissociation and culturing in DMEM/F12 serum-free medium containing 2 mM glutamine, 0.6% glucose, 9.6 g/mL putrescine, 6.3 ng/mL progesterone, 5.2 ng/mL sodium selenite, 0.025 mg/ml insulin, and 0.1 mg/mL transferrin sodium salt (Sigma-Aldrich, St. Louis, MO, USA), supplemented with EGF and bFGF. GSC lines grown as floating spheres in serum-free medium supplemented with mitogens showed an undifferentiated state, as indicated by their rounded morphology, high nuclear/cytoplasm ratio. For differentiation, GSC lines were grown in medium supplemented with 5% fetal bovine serum (FBS, EuroClone, Milan, Italy). Human GSC lines were authenticated by short tandem repeat (STR) profiling according to the American National Standards Institute/American Type Culture Collection Standard ASN-0002-2011.12 using the Cell line Integrated Molecular Authentification database (CLIMA),13 and Cellosaurus STR database (CLASTR) of the Cellosaurus database (ExPASy) at the IRCC Ospedale Policlinico San Martino, Interlab Cell Line Collection (ICLC), Biological Resource Center (CRB-HSM), Genova, Italy [30]. All cell lines were isocitrate dehydrogenase (IDH) 1/2 wild-type.

### 2.2. Antibodies

The following mouse monoclonal antibodies (mAbs) were used: anti-glyceraldehyde-3-phosphate dehydrogenase (GAPDH, Santa Cruz Biotechnology, Heidelberg, Germany) and anti-transient receptor potential cation channel A1 (TRPA1) (Santa Cruz Biotechnology), anti-class III-beta-tubulin (β_III_-tubulin) (Millipore, Billerica, MA, USA), anti-Aml1 (Cell Signaling Technology, Danvers, MA, USA). The following polyclonal Abs were used: rabbit anti-glial fibrillary acidic protein (GFAP) and rabbit anti-transient receptor potential vanilloid 1 (TRPV1) (ThermoFisher Scientific, Rodano, Italy). The following secondary Abs were used: horseradish peroxidase (HRP)-conjugated sheep anti-mouse IgG and HRP-conjugated donkey anti-rabbit IgG (Cell Signaling Technology), anti-mouse Alexa Fluor-488 (ThermoFisher Scientific), anti-rabbit Alexa Fluor-594 (ThermoFisher Scientific), PE-conjugated anti-rabbit (Santa Cruz Biotechnology), PE-conjugated anti-mouse (Santa Cruz Biotechnology). The following isotypes were used: PE-conjugated mouse IgG1 and PE-conjugated rabbit IgG1 (ThermoFisher Scientific).

### 2.3. Western Blot Analysis

Total proteins from GSC lines were obtained as previously described [31]. Twenty μg of the lysate was separated on a SDS-polyacrylamide gel, transferred onto Hybond-C extra membranes (GE Healthcare, Milan, Italy), blocked with 5% low-fat dry milk in PBS-Tween 20, immunoblotted with anti-GFAP (1:300), anti-β_III_-tubulin (1:1000), anti-Aml1 (1:1000), anti-TRPV1 (1:1000) and anti-TRPA1 (1:500) followed by the appropriate HRP-conjugated secondary Abs (1:2000). For quantification, anti-GAPDH (1:1000) Ab was used as the loading control. The detection was performed using the Lite-Ablot PLUS or the Lite-Ablot TURBO (EuroClone) kits and densitometric analysis was carried out by a Chemidoc using the Quantity One software (BioRad, Milan, Italy).

### 2.4. Cell Death Analysis

GSC cell differentiation was induced as previously published [28,31]. Undifferentiated (GSC#1 and GSC#83) and differentiated (D-GSC#1 and D-GSC#83) lines were treated with TRPV1 agonist, capsaicin (CPS, 50 and 200 μM, Sigma-Aldrich) or TRPA1 agonist, trans-Cinnamaldehyde (CINN, 50 and 100 μM, Sigma-Aldrich) for 24 h. Then, cell death was evaluated using FITC-conjugated Annexin V and PI staining, followed by cytofluorimetric and FACS analysis. Briefly, cells were incubated with 5 μL Annexin V-FITC or 20 μg/mL PI (ThermoFisher Scientific) for 10 min at room temperature. The cells were then analyzed by flow cytometry using CellQuest software (4.0, Becton Dickinson, San Jose, CA, USA).

### 2.5. Confocal Laser Scanning Microscopy Analysis

For immunophenotyping, GSC and D-GSC lines were treated and fixed as previously published [31]. Then, cells were incubated with 5% of bovine serum albumin (BSA, Sigma-Aldrich) and 0.1% of Tween-20 in PBS for 1 h at room temperature and then stained with anti-human TRPV1 Ab (1:1000) overnight at 4 °C. Then, samples were washed with 0.3% of Triton X-100 in PBS, incubated with Alexa Fluor 594-conjugated secondary Ab for 1 h at 37 °C. In co-labeling experiments, cells were also stained with anti-human TRPA1 Ab (1:50) overnight at 4 °C. Finally, samples were washed with 0.3% of Triton X-100 in PBS, incubated with Alexa Fluor 488-conjugated secondary Ab for 1 h at 37 °C. Cells were also co-labeled with anti-β_III_-tubulin and GFAP Abs.

As described above, cells were incubated with 5% of BSA and 0.1% of Tween-20 in PBS for 1 h at room temperature and then stained with anti-β_III_-tubulin Ab (1:50) overnight at 4 °C. Then, samples were washed with 0.3% of Triton X-100 in PBS, incubated with Alexa Fluor 488-conjugated secondary Ab for 1 h at 37 °C. Cells were also stained with anti-GFAP Ab (1:500) overnight at 4 °C. Finally, samples were washed with 0.3% of Triton X-100 in PBS, incubated with Alexa Fluor 594-conjugated secondary Ab for 1 h at 37 °C. Then, nuclei were stained with 4′,6-diamidino-2-phenylindole (DAPI, BioRad). Slides were then analyzed with C2 Plus confocal laser scanning microscope (Nikon Instruments, Firenze, Italy). Optimized emission detection bandwidths were configured by Zeiss Zen control software.

### 2.6. Gene Expression Analysis

Total RNA was extracted with the RNeasy Mini Kit (Qiagen, Milan, Italy), and cDNA was synthesized using the High-Capacity cDNA Archive Kit (Applied Biosystems, Foster City, PA, USA) according to the manufacturer’s instructions. Quantitative RT-PCR (qRT-PCR) was performed by using the IQ5 Multicolor real-time PCR detection system (BioRad). The reaction mixture contained the Advanced Universal SYBRGreen Supermix (BioRad). Human *TRPV1*, *TRPA1*, *Aml1b*, *Aml1c* and *GAPDH* RT^2^qPCR Primer assay (Qiagen) were used. The PCR parameters were 10 min at 95 °C followed by 40 cycles at 95 °C for 15 s and 60 °C for 40 s. All samples were assayed in triplicate in the same plate. The relative amount of target mRNA was calculated by the 2^−ΔΔCt^ method, using *GAPDH* as a housekeeping gene.

### 2.7. qRT-PCR and PCR Array

The Human Stem Cell Signaling Pathway Finder PCR Arrays with related reagents were purchased from SABiosciences (Frederick, MD, USA). Total RNA was extracted from GSC and D-GSC lines. Two μg of total RNA from each sample were subjected to reverse transcription in a total volume of 20 μL using the Reaction Ready First Strand cDNA (SABiosciences). cDNAs were analyzed by qRT-PCR performed using a IQ5 Multicolor Real-Time PCR Detection system, the Super Array’s RT^2^ real-time SYBR Green PCR Master Mix and the relative array, according to manufacturer’s instructions. Measurement of two housekeeping genes (*GAPDH*; *Ribosomal protein*, *large*, *P0*; *RPLP0*) on the samples was used to normalize mRNA content. Data acquisition was performed using the web-based integrated PCR Array Data Analysis Tem-235 plate provided by SABiosciences.

### 2.8. Statistical Analysis

The data presented represent the mean and standard deviation (SD) of at least three independent experiments. The statistical significance was determined by analysis of variance (ANOVA) or Student’s *t*-test; * *p* < 0.01.

## 3. Results

### 3.1. Expression of the Aml1 Spliced Variants and TRP Channels in NSC and D-NSC

**Figure 1:**
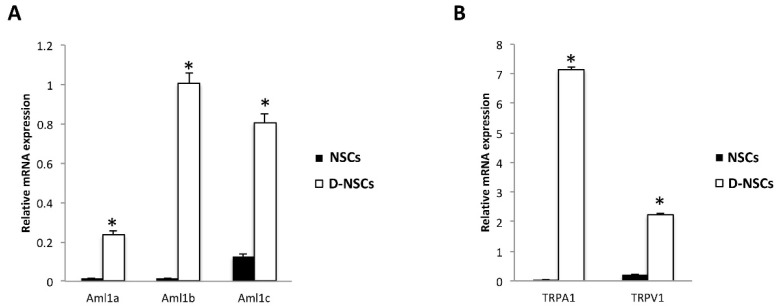
Phenotype characterization of NSCs: (**A**) The relative *Aml1* isoforms mRNA expression in NSCs (NSC#1 and NSC#2) and D-NSCs (D-NSC#1 and D-NSC#2) evaluated by qRT-PCR. (**B**) The relative *TRPA1* and *TRPV1* mRNA expression in NSCs (NSC#1 and NSC#2) and D-NSCs (D-NSC#1 and D-NSC#2) evaluated by qRT-PCR. *AML1*, *TRPA1* and *TRPV1* mRNA levels were normalized for *GAPDH* expression. Data are expressed as mean ± SD.* *p* < 0.001 vs. NSCs.

The expression of *Aml1a*, *Aml1b* and *Aml1c* isoforms of *Aml1/Runx1* were investigated in the NSC#1 and NSC#2 by qRT-PCR. As shown in Figure 1A, mRNA for *Aml1a*, *Aml1b* and *Aml1c* was evidenced in the NSCs; the differentiation of NSCs for 14-days (D-NSCs) strongly increased the expression of all *Aml1* variants, compared to NSCs, with *Aml1b* showing the higher fold increase (62-fold).

The transcriptional factor Runx1/Aml1 regulates the expression of many ion channels and receptors, including some members of the TRP thermal and chemical receptors [21]. Thus, the expression of the *TRPA1* and *TRPV1* channels, belonging to the anchyrin and vanilloid TRP subfamilies, respectively, was evaluated in NSC#1 and NSC#2 and D-NSC#1 and D-NSC#2. Transcripts for *TRPV1* and *TRPA1* were found in both NSC#1 and NSC#2 and their expression markedly increased in D-NSC#1 and D-NSC#2, compared to NSC#1 and NSC#2, respectively, with *TRPA1* showing the higher increase (164-fold) (Figure 1B).

### 3.2. Overexpression of Aml1b and Aml1c Variants and TRP Channels in Distinct PN- and MES-like GSCs Compared to NSCs

We previously reported the expression of Aml1a in GSCs and the role of the TRPV2 agonist, CBD, in mediating its expression and functions [28].

Herein, the expression of *Aml1b* and *Aml1c* splice variants was analyzed at mRNA level by qRT-PCR in seven different GSC lines, with PN- (GSC#1, #23C, #28, #68 and #70), and MES-like phenotype (GSC#30 and GSC#83) [14,15,32]. An *Aml1b* and *Aml1c* mRNA upregulation, although at different levels, was observed in all the GSCs analyzed, compared with NSCs. The *Aml1b*, with respect to the *Aml1c* isoform, showed the higher mRNA increase in MES-like GSC#83 and GSC#30 lines, respectively, compared with NSCs (Table 1). The higher *Aml1c* mRNA expression was evidenced in MES-like GSC#83, whereas lower mRNA expression was observed in the PN-like GSC#68 line, compared with NSCs (Table 1).

In regard to TRP channels, the *TRPA1* mRNA expression was increased in all the GSC lines, independently of the phenotype, with the exception of the GSC#23C cells expressing lower levels (Table 2). Higher *TRPA1* values, compared to NSCs, were found in MES-like GSC#83; the *TRPV1* expression was mainly increased in GSC#83 and strongly downregulated in PN-GSC lines, except in GSC#70 cell line, compared to NSCs (Table 2).

Overall, our results showed a marked overexpression of *Aml1b* and *Aml1c* variants, compared with NSCs in all the PN- and MES-GSC lines; moreover, a marked increase of *TRPA1* and *TRPV1* mRNA was demonstrated in MES-GSCs as well as a marked reduction of *TRPV1* expression in PN-GSCs with respect to NSCs.

### 3.3. Differentiation of GSC Lines Increases the Aml1b/Aml1c and TRP Ion Channel mRNA Levels

The expression of *Aml1b* and *Aml1c*, as well as *TRPA1* and *TRPV1*, was investigated during GSCs differentiation. The differentiation of PN- and MES-GSCs for 14 days (D-GSCs) resulted in the cells showing glial morphology (Figure S1 in Supplementary Materials) [28,31], and, as shown in Table 3, the *Aml1b* and *Aml1c* mRNA levels were upregulated in all D-GSCs compared with the respective GSCs with the main increase in MES-like D-GSC#30 line (Table 3). This enhancement was even more evident when comparing D-GSCs with D-NSCs (Table S1).

In addition, *TRPA1* mRNA expression was mainly increased in the MES-like D-GSC#83 line and was reduced in D-GSC#28, #68 and #70 PN-GSC lines, compared to GSCs. Conversely, *TRPV1* mRNA expression decreased in both PN- and MES-like D-GSCs (Table 4). This enhancement was even more evident when comparing D-GSCs with D-NSCs (Table S2).

### 3.4. TRPA1 and TRPV1 Channel Expression in PN- and MES-like GSCs and D-GSC Lines

**Figure 2:**
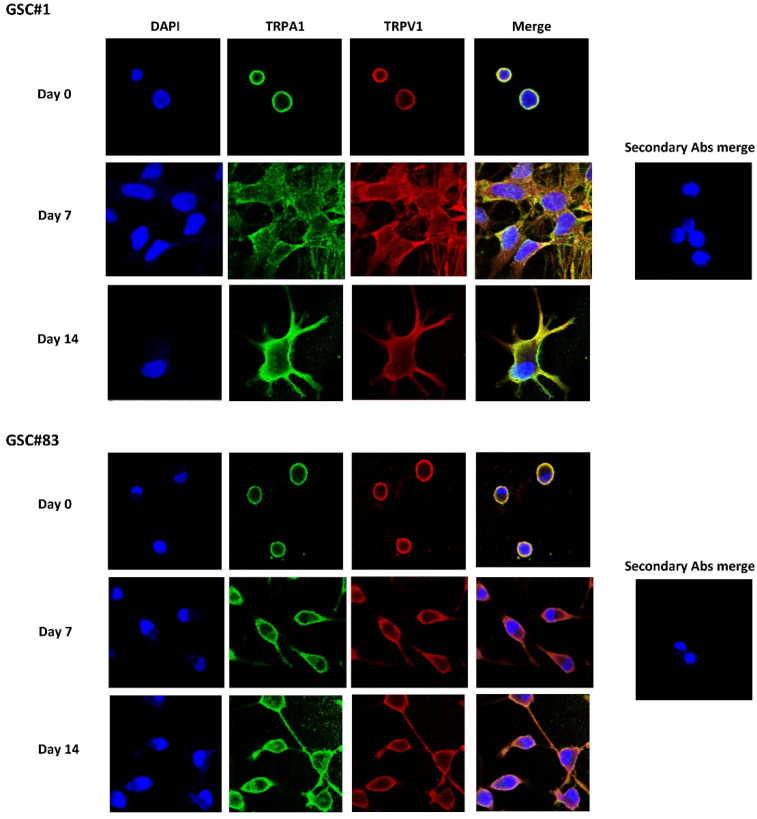
TRPs expression in GSCs during differentiation. GSCs (day 0) and D-GSCs (day 7 and 14) were fixed, permeabilized, and stained with anti-human TRPA1 and TRPV1 Abs followed by Alexa Fluor-488 and Alexa Fluor-594 secondary Abs, respectively. 40,6-diamidino-2-phenylindole (DAPI) was used to counterstain nuclei. Magnification 60×.

Among the seven different GSC lines, we studied the GSC#1 and GSC#83 lines, as representative cell lines previously well characterized for the expression of a proneural and mesenchymal phenotype [14,32] (Figure S1). In these cell lines, the expression of TRPA1 and TRPV1 proteins was evaluated by confocal microscopy and western blot analyses. Data showed that TRP channels are expressed in both cell lines and, partially colocalized in the plasma membrane in GSCs and during the differentiation (Figure 2).

### 3.5. ERK Phosphorylation Increases the Aml1b and Aml1c Protein Expression and Induces Changes in TRP Channel Expression during GSC Differentiation

**Figure 3:**
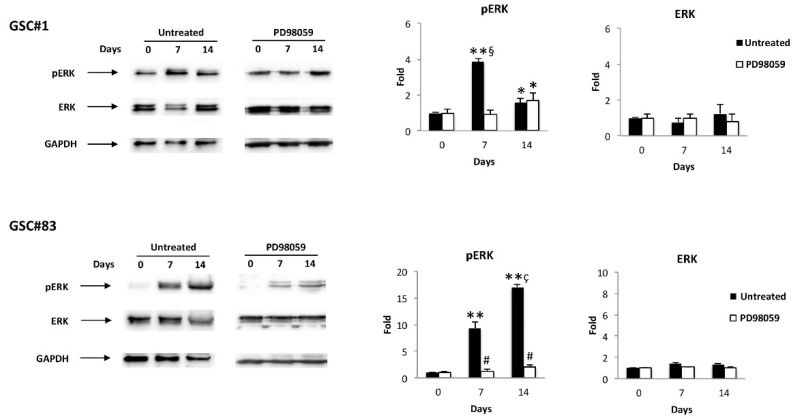
ERK modulation during GSC differentiation: western blot analysis of pERK and ERK protein levels in GSC#1 and GSC#83 untreated or treated with PD98059 for up to 14 day of differentiation. Blots are representative of one of three separate experiments. ERK densitometry values were normalized to GAPDH used as loading control. The pERK levels were determined with respect to ERK levels. * *p* < 0.05 vs. undifferentiated cells (time 0); ** *p* < 0.001 vs. undifferentiated cells (time 0); ^#^ *p* < 0.001 vs. untreated cells; ^ç^ *p* < 0.001 vs. 7 days of differentiation; ^§^ *p* < 0.001 vs. 14 days of differentiation.

The ERK pathway has been found to regulate proliferation and differentiation in GBM [33,34] and GSCs [35]. Thus, the level of pERK and total ERK was evaluated in GSC#1/D-GSC#1 and GSC#83/D-GSC#83 lines at different times of differentiation, by western blot, using an anti-pERK and anti-ERK mAbs. ERK was basally phosphorylated in GSC#1 and its phosphorylation level increases at seven days of differentiation, then declines at later time-point (14 days) (Figure 3); no ERK phosphorylation was evident at basal level in GSC#83; ERK phosphorylation was induced at day 7 and progressively increased until day 14 of differentiation (Figure 3). To determine ERK involvement, the GSC#1 and GSC#83 were treated for 24 h at different times of differentiation with the MEK1 inhibitor, PD98059 (50 μM), able to completely inhibit ERK phosphorylation in both GSC lines at any time tested (Figure 3).

**Figure 4:**
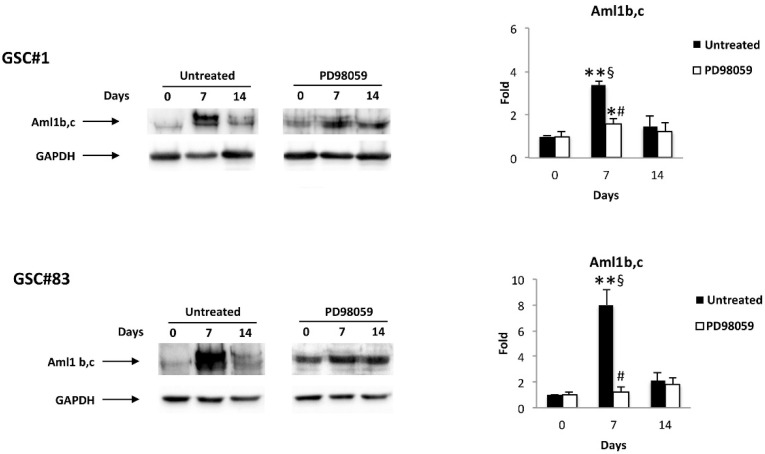
Aml1 splice variant modulation during GSC differentiation: western blot analysis of Aml1b and Aml1 c protein levels in GSC#1 and GSC#83 untreated or treated with PD98059 for up to 14 day of differentiation. Blots are representative of one of three separate experiments. Aml1 b/c densitometry values were normalized to GAPDH used as loading control. * *p* < 0.05 vs. undifferentiated cells (time 0); ** *p* < 0.001 vs. undifferentiated cells (time 0); ^#^ *p* < 0.001 vs. untreated cells; ^§^ *p* < 0.001 vs. 14 days of differentiation.

Thus, we evaluated if ERK activation was capable of affecting the Aml1b and Aml1c protein expression during GSC#1 and GSC#83 differentiation by using an anti-Aml1 mAb, which recognizes all the Aml1 splice variants. Parallelly to ERK phosphorylation kinetic, the Aml1b and Aml1c protein levels increase at day 7 of differentiation (mainly in GSC#83) and thereafter declines at day 14 (Figure 4). Finally, the enhancement of Aml1b and Aml1c protein levels at seven days of differentiation was reverted by the treatment with the PD98059 inhibitor in both GSC#1 and GSC#83 lines (Figure 4)

**Figure 5:**
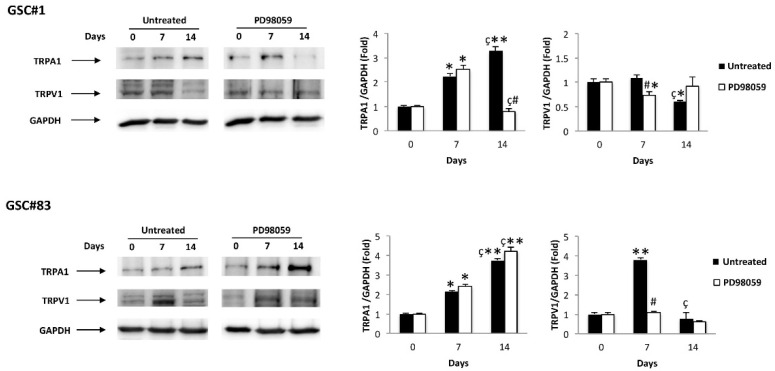
TRP modulation during GSC differentiation: western blot analysis of TRPA1 and TRPV1 protein levels in GSC#1 and GSC#83 untreated or treated with PD98059 for up to 14 day of differentiation. Blots are representative of one of three separate experiments. TRPA1 and TRPV1densitometry values were normalized to GAPDH used as loading control. * *p* < 0.01 vs. undifferentiated cells (time 0); ** *p* < 0.001 vs. undifferentiated cells (time 0); ^#^ *p* < 0.001 vs. untreated cells; ^ç^ *p* < 0.01 vs. 7 days of differentiation.

In addition, in the same manner of ERK phosphorylation, TRPV1 mRNA levels increased at 7 days in both GSCs during differentiation, and this upregulation was reduced, mainly in GSC#83, by the PD98059 inhibitor. On the contrary, PD98059 reduces the TRPA1 upregulation at 14 days of differentiation in PN-like D-GSC#1, whereas increased TRPA1 levels were found in MES-like GSC#83 (Figure 5), suggesting that in this GSC, the enhanced TRPA1 protein expression is ERK-independent.

### 3.6. Specific TRPA1 and TRPV1 Agonists Stimulate Apoptotic/Necrotic Cell Death in PN- and MES-GSCs and D-GSCs

**Figure 6:**
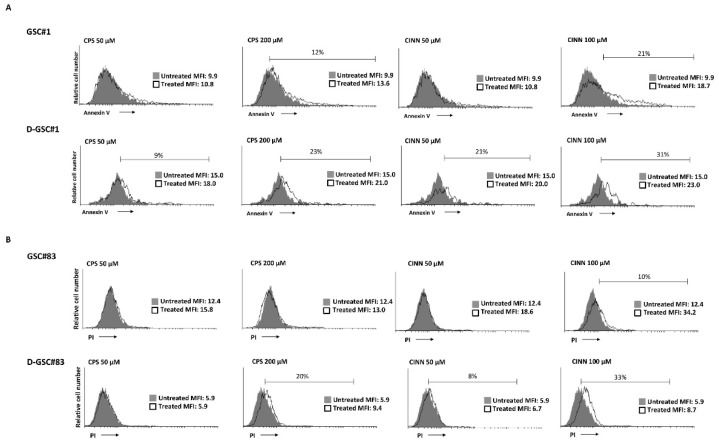
Phenotype characterization of NSCs: (**A**) Annexin V staining was analyzed by flow cytometry in in GSC#1 and D-GSC#1 at 7 days of differentiation, treated with CPS (50 and 200 μM) or CINN (50 and 100 μM) for 24 h. A representative one out of three experiments have been shown. Percentages represent the positive cells. MFI = mean fluorescence intensity. (**B**) The cytotoxic effects in GSC#83 and D-GSC#83 at 7 days of differentiation, treated with CPS (50 and 200 μM) or CINN (50 and 100 μM) were determined by PI staining and cytofluorimetric analysis assay. A representative of three experiments has been shown. Percentages represent the positive cells. MFI = mean fluorescence intensity.

We then analyzed whether the increased TRPA1 and TRPV1 expression at day 7 of differentiation corresponded to an increased functionality of these receptors. Thus, we determined the sensitivity of GSCs to the TRP agonists-induced cell death. GSC#1 and GSC#83 cells were treated with different doses of the TRPA1 agonist, trans-Cinnamaldehyde (CINN) [36] and TRPV1 agonist, capsaicin (CPS) [37] for 24 h. The percentage of dead cells was evaluated by staining the GSCs with PI or FITC-conjugated Annexin V and cytofluorimetric analysis. We found that PN-like GSC#1 line was sensitive to high CINN and CPS doses, with 21% and 12% of apoptotic Annexin V-positive cells, respectively. Seven days differentiation increases the GSC#1 sensitivity, as shown by the enhancement in Annexin V-positive cells both at low and high CINN and CPS doses (Figure 6A). No PI-positive cells were evidenced in CINN/CPS-treated GSC#1 and D-GSC#1 (data not shown). Regarding the GSC#83 line, these cells were completely resistant to the CPS cytotoxic effects and only 10% of 100 μM CINN-treated cells were PI-positive necrotic cells. Differentiation increases the sensitivity to CPS treatment (20% PI-positive cells at 200 μM of CPS) and for both doses of CINN (8% and 33% of PI-positive necrotic cells upon 50 and 100 μM treatment, respectively) (Figure 6B). No Annexin V-positive cells were evidenced in CPS or CINN-treated GSC#83 and D-GSC#83 (data not shown).

Overall, GSC#1 are more sensitive compared to GSC#83 to CINN and CPS-induced cytotoxic effects. GSC differentiation increases the sensitivity to CINN or CPS treatment mainly in PN-like GSC line.

### 3.7. NGF Levels Increase during GSC Differentiation

**Figure 7:**
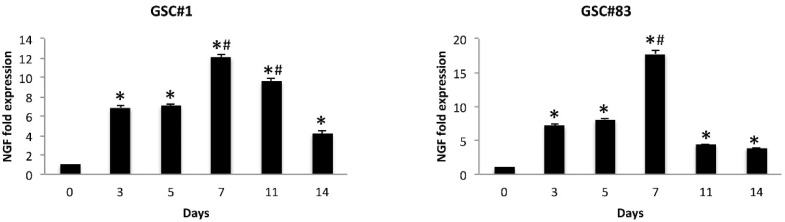
*NGF* modulation during GSC differentiation. Relative *NGF* mRNA expression during GSCs differentiation was evaluated by qRT-PCR. *NGF* mRNA levels were normalized for *GAPDH* expression and were expressed as relative fold with respect to the corresponding GSC lines used as calibrator. * *p* < 0.001 vs. undifferentiated NSCs; ^#^ *p* < 0.01 vs. 3, 5, 11, 14 days of differentiation.

It was demonstrated that the effect of ERK phosphorylation depends on activation of the NGF pathway [38,39]. Thus, *NGF* was evaluated at mRNA levels in GSCs and D-GSCs of both PN and MES phenotypes at different times during differentiation (3, 5, 7, 11 and 14 days) by qRT-PCR. We found that the mRNA levels of *NGF* progressively increase until day 7 and decline thereafter, although with different kinetic in both GSC#1 and GSC#83 (Figure 7).

### 3.8. GSC Differentiation Is Accompanied by Changes of Gene Pathways Involved in GSC Differentiation

The modulation of *Aml1b/c* mRNA and protein expression during the GSC differentiation is associated with changes of different genes for intracellular signals. Thus, the expression of 84 genes involved in signal transduction pathways important for stem cell maintenance, proliferation and/or differentiation, in PN-like GSC#1and MES-like GSC#83lines in proliferative condition and at seven days of differentiation, was evaluated by a Stem Cell Signaling Pathway Finder PCR array. 

The results showed that 19 (10 upregulated and nine downregulated) and 14 (seven upregulated and seven downregulated) genes belonging to the *TGFβ/Smad* (*BMP1RB*), *CDX2*, *SMAD2*, *SMAD3*, *SMAD9*, *TGFBR2*, *FGF* (*FGFR2*), *NFAT* (*NFAT5*), *Wnt/β-catenin* (*FZD1*, *FDZ3* and *FDZ9*) and *NOTCH* (*NOTCH-3* and *-4*) were modulated in GSC#1 and GSC#83 at day 7 of differentiation, respectively (Table 5 and Table 6). These findings suggest that the modulation of *Aml1b/Aml1c* in GSCs induced by ERK activation is a part of a differentiative network that controls the genetic transcriptional program of GSC differentiation.

## 4. Discussion

Malignant brain tumors, including GBM, are known for their high degree of cellular heterogeneity, invasiveness, aggressiveness and lethality. GSCs are crucial for the malignancy of GBM and likely represent the consequence of transformation of the normal NSC compartment [40]. The GSCs respond to differentiation signals both at transcriptional and post-transcriptional levels. Among the transcriptional factors, Aml1/Runx1 is thought to be involved in the control of the balance between cell proliferation and differentiation [41], and the cellular decision to continue proliferating or to differentiate depends on the relative expression levels of the Aml1 variant forms [42]. We previously reported that Aml1a isoform is involved in GSCs proliferation, stemness and pluripotency, and differentiation [28].

Herein, by qRT-PCR analyses, we found that NSCs express the *Aml1* splice variants of *Aml1/Runx1* and the downstream TRP ion channel genes, *TRPA1* and *TRPV1*, with *Aml1c* and *TRPV1* showing the higher expression. Serum-induced differentiation enhances the *Aml1a*, *Aml1b* and *Aml1c* and *TRPV1* mRNA expression, whereas *TRPA1* was reduced in D-NSCs. A significant amount of data on *Aml1/Runx1* mutations and cancers and the relationship between *Aml1/Runx1* overexpression and poor prognosis have been reported [43,44]. An Aml1/Runx1 protein accumulation, correlating to tumor aggressiveness has been reported in astrocytomas [45]. In addition, a marked accumulation of wild-type *Aml1b* variant, and of a novel *Aml1b* isoform lacking the exon 6 in ovarian cancer patients has been reported, which correlates with poor patient outcomes [46].

In this paper we analyzed seven different GSC lines having a PN- or MES-like phenotype. We found a strong overexpression of both *Aml1b* and *Aml1c* variants in all the PN- and MES-GSC lines compared to NSC. The increase of *TRPA1* and *TRPV1* was more evident in MES-GSCs. Instead, the reduction of *TRPV1* mRNA expression was evidenced mainly in PN-GSCs, compared to NSCs. Serum-induced GSC differentiation enhanced the *Aml1b* and *Aml1c* levels in D-GSC lines, compared to GSCs. Comparative analysis of *Aml1b* and *Aml1c* mRNA expression in D-GSCs vs. GSCs or D-NSC confirms the substantial increase of *Aml1b/Aml1c* levels, more evident in the GSC#30 and GSC#83 MES lines, and a reduction of *Aml1c* in D-GSC#68 and #70 PN-GSC lines. The analysis of the expression of Aml1b and Aml1c variants at protein levels in GSCs and D-GSCs evaluated by western blot analyses evidenced that both Aml1b/c proteins accumulate at day 7 and are sustained upon 14 days of differentiation.

The differentiation program of GSCs is accompanied by the change of *TRPA1* and *TRPV1* expression compared to D-NSC. High levels of *TRPA1* were found in NSCs and their expression decreased in D-NSCs; on the contrary, increased *TRPA1* mRNA expression in MES-D-GSCs, whereas similarly to NSCs a reduction of *TRPA1* and *TRPV1* mRNA expression was reported in the majority of the D-GSCs.

TRPA1 is mainly expressed in primary sensory neurons, but it has been also described in non-excitable cells, including those derived from neural crest stem cells, such as glial cells and oligodendrocytes and astrocytes. In pain-sensitive nerve endings, TRPA1 senses the oxidative stress and transduces it into neural signals [47]; similarly, in GBM, oxidative stress activates TRPA1 channels [48]. Chemical stimulation of TRPA1 seems to activate stem cells [49] and at least in skeletal muscle, TRPA1 agonists stimulate migration and early stem cell differentiation [50]. Moreover, recently, a crosstalk between Runx1 and TGFβ signaling pathway regulating the *TRPA1* and *TRPV1* expression and an increased Ca^2+^ activity was evidenced during megakaryocyte differentiation and silencing of Aml1/Runx1 markedly reduced the *TRPA1* and *TRPV1* mRNA expression [51]. In addition, in lung adenocarcinoma, direct binding of TRPA1 to FGFR2 resulted in a constitutive receptor activation, thereby promoting tumor progression [52].

Regarding *TRPV1*, it is expressed in NSCs and its level increases in D-NSCs. The MES-like GSCs express high *TRPV1* mRNA levels, whereas a reduced *TRPV1* mRNA levels was evidenced in PN-like GSC and D-GSC lines. It was already demonstrated that *TRPV1* is expressed in neural precursor cells (NPCs) [53] and it plays a major role in NSC survival [54] and in retinoid acid-induced neuron differentiation [55]. *TRPV1* loss promotes proliferation of NPCs in vivo and in vitro. Knockout of *TRPV1* increases post-natally proliferating cells in stem cell niches (e.g., the dentate gyrus and SVZ). Under mild differentiating conditions in vitro, NPCs from *TRPV1* knockout mice express stem cell genes and at less extend GFAP and β_III_-tubulin differentiation markers. However, no stemness reduction was evidenced in NPCs by *TRPV1* loss. More importantly, NPCs migrate into high-grade astrocytomas to reduce glioma expansion and prolong survival by release of fatty acid ethanolamides by triggering a TRPV1-dependent astrocytoma death, via the activation of ATF3 of the ER stress pathway [56]. On this basis, we can hypothesize that *TRPV1* loss in differentiated GSCs, which is different from high TRPV1 expression in D-NSC, represents an escape mechanism of tumor cells from death mediated by TRPV1 agonists. Moreover, the correlation between the expression of a most stable *TRPV1 variant-3 (TRPV1v3)* in GSCs and survival suggests an important role played by TRPV1 in glioma [57]. Similarly, we also found that the higher expression of *TRPV1* and *TRPA1* in D-GSCs is associated to CPS- and CINN-induced cell death, mainly in PN-like D-GSC line. 

The transcriptional activity of Aml1/Runx1 is regulated by controlling the MAPK-mediated phosphorylation of Aml1/Runx1. Activated ERK enters the nucleus and promotes the clustering of transcription factors, including Aml1/Runx1, to function. ERK-dependent phosphorylation of Ser-249 and -266 disrupts the interaction of Runx1 with the mSin3A transcriptional corepressor and potentiates the transactivation and the transforming abilities of Aml1/Runx1 [58]. ERK signaling controls the neural genetic programs in cortical development and gliomagenesis [33,34,59]. ERK signaling does not promote neoplastic proliferation alone, but influences neural cell fate selection. On the other hand, ERK is commonly activated in glioma and glioneuronal tumor, raising the question of how this pathway differentially influences oncogenic cell fates. In NSCs, ERK phosphorylation inhibited differentiation [59]. Herein, we found that treatment of D-GSC lines with the MEK1 inhibitor PD98059 reverted the accumulation of Aml1b and Aml1c proteins at seven days of differentiation.

Similarly, TRPV1 protein levels increased at seven days in both the PN- and MES-like GSCs, in an ERK-dependent manner. In accordance, CPS-induced ERK activation is reversed by the TRPV1 antagonist, capsazepin or MEK inhibitor [60,61,62], and CPS triggered ERK phosphorylation in sensory neurons [63]. The increase of TRPA1 protein expression observed during PN-like GSC#1 was inhibited by PD98059 at 14 days of differentiation. Instead, in MES-like GSC#83, PD98059 did not revert the TRPA1 increase, suggesting that this event is ERK-independent. In this regard, a role for p38MAPK in *TRPA1* mRNA increase in trigeminal ganglia and sensory neurons has been reported [64]; moreover, intrathecal administration of the p38MAPK inhibitor, SB203580 reduced the *TRPA1* mRNA levels in DRGs [65].

NGF signaling is essential for the sustained expression of Runx1. Runx1 induction in olfactory neuroblastoma cell line JFEN is associated with TrkA expression, and this correlates with specific in vivo expression of Runx1 and TrkA in DRG nociceptive neurons [66]. A *Runx1/Aml1* mRNA accumulation found in astrocytomas, correlates with tumor aggressiveness [45]. In developing neurons, binding of NGF to its receptor TrkA triggers receptor dimerization, autophosphorylation, and MAPK signaling pathway activation [67]. NGF has been reported to increase GBM cell growth via TrkA receptor phosphorylation [68] and ERK promotes the proliferation and inhibits neuronal differentiation of NSC [59]. Finally, NGF has been found to increase the *TRPA1* mRNA expression in a p38MAPK-dependent manner [64]. Overall, our data are in agreement with previously reported data. We found in both GSC#1 and GSPC#83 lines that *NGF* mRNA levels progressively increase until day 7, and decline thereafter. Thus, it seems possible that the binding of NGF to TrkA receptors triggers ERK phosphorylation in GSCs that stimulates the increase of Aml1b/Aml1c proteins.

Runx1 contributes to the acquisition of aggressive and invasive phenotype in MES- and PN-like GSC phenotypes in a TGFβ-dependent manner [69,70]. In this regard, both PN- and MES-like GSC differentiation modulates the TGFβ/Smad and Wnt/βFGF signaling pathways. Thus, increased *anti-Mullerian hormone (AMHR2)* and *Bone morphogenetic protein (BMPR2* and *BMPR1B*) mRNA expression was evidenced in both D-GSCs at 7 days of differentiation compared to GSCs. AMHR2 is a potent regulator of TGFβ/BMP signaling; AMHR2 hetero-dimerizes with ACVR1, regulates the BMPR2- and BMPR1B-mediated differentiation signals and SMAD survival signaling [71]. In this regard, reduced *SMAD3* and increased *SMAD9* and *SMAD2* mRNA expression were evidenced in GSC#83 and GSC#1, respectively. The *caudal-type homebox 2 (CDX2)* gene was increased in both GSCs. CDX2 promotes the malignant behavior of glioma cells; *CDX2* knockdown suppresses glioma cell proliferation, migration and invasion and facilitates the apoptosis of glioma cells [72]. 

The notch pathway seems to be downregulated during GSCs differentiation, with GSC#83 and GSC#1 cells downregulating the *Notch4* and *Notch3,* and *Notch4* expression, respectively [73]. The Wnt signaling pathway controls neurogenesis from NSCs by regulating maintenance, proliferation and differentiation of progenitor cells into newborn neurons. Here, we found that *FZD1*, *FZD3* and *FDZ9* were increased in D-GSC#1 and D-GSC#83, respectively, compared to GSCs. *FZD1* is highly expressed in NSCs [74] and its knockdown decreases neuron differentiation and increases astrocyte generation; FZD3 is associated with non-canonical β-catenin-independent signaling, and stimulates the neural crest formation, suggesting a role in cell fate commitment [75].

*IL-6* expression increases with glioma malignancy grade and is associated with shorter survival of GBM patients. IL-6 and LIF are crucial mediators of self-renewal capacity of GSC [76]. Herein, we found that in GSC#83, which increases the expression of the *CD133* stem cell marker in D-GSC#83 [14], thus the *IL-6/LIFR* levels were increased, whereas the reduction of *LIFR* levels was observed in GSC#1, showing *CD133* reduction in D-GSCs, in comparison to GSCs. 

Moreover, increased levels of *immunoglobulin kappa J region (RBPJ)* mRNA, which contributes to GBM malignancy and promotes proneural–mesenchymal transition via the IL-6/STA3 pathway [77], were evidenced in MES-like GSC#83 cells, suggesting that mesenchymal GSC#83 may be derived from a GSC with a proneural phenotype [15]. In the GBM stem cell compartment, the proneural–mesenchymal transition, the equivalent of the epithelial–mesenchymal transition associated with aggressive cancers, under the selective pressure of treatment agents is responsible for the acquisition of multitherapy resistance phenotype [78]. Finally, due to their role in promoting the cell cycle of gliomas, *NFAT5* and *E2F5* TFs are downregulated in seven days for the D-GSC#83 and D-GSC#1 lines, respectively [79].

## 5. Conclusions

The overexpression of *Aml1b* and *Aml1c* splice variants of the Runx1/Aml1 transcriptional factor was detected in GSC lines, compared to NSCs. GSC heterogeneity was also evidenced, with GSCs mainly expressing a proneural or a mesenchymal-like phenotype. Even if we analyzed only one GSC cell line for each phenotype, our data suggest that GSC differentiation accompanied by ERK phosphorylation is able to induce: (a) accumulation of Aml1b and Aml1c proteins, (b) changes in the downstream TRPA1 and TRPV1 ion channels expression and consequently in the TRP agonists-mediated cytotoxic functions, (c) increase in *NGF* mRNA levels, (d) activation of distinct TGFβ/Smad and Wnt/β-catenin and bFGF signaling pathways in proneural- and mesenchymal-like GSCs. Thus, the need to increase the knowledge on the intercellular signals controlling GSC differentiation may shed light on GSC clonal heterogeneity and improve the development of more specific target therapy in GBM patients.

## Figures and Tables

**Table 1:** *Aml1b* and *Aml1c* splice variants mRNA expression in GSCs.

Phenotype	Cell Lines	*Aml1b* mRNA		*Aml1c* mRNA	
		Relative mRNA Expression ± SD	Fold Expression	Relative mRNA Expression ± SD	Fold Expression
	NSC	0.016 ± 0.001	1.00	0.124 ± 0.012	1.00
PN-like	GSC#1	1.141 ± 0.031	70.34	0.963 ± 0.089	7.77
MES-like	GSC#83	2.432 ± 0.101	150.23	4.626 ± 0.651	37.31
PN-like	GSC#23C	0.721 ± 0.032	45.00	0.831 ± 0.021	6.70
PN-like	GSC#28	0.733 ± 0.034	45.62	0.923 ± 0.023	7.44
MES-like	GSC#30	1.952 ± 0.102	107.56	0.322 ± 0.004	2.60
PN-like	GSC#68	1.721 ± 0.111	121.87	0.214 ± 0.005	1.69
PN-like	GSC#70	1.232 ± 0.055	78.87	0.324 ± 0.013	2.61

mRNA samples extracted from NSCs and GSCs. Data represents relative mRNA levels and fold expression of individual gene in GSCs compared to the NSCs. The expression levels were normalized to the average Ct value of two housekeeping genes (*GAPDH* and *RPLP0*) and calculated by the 2^−ΔΔCt^ method.

**Table 2:** *TRPA1* and *TRPV1* channels mRNA expression in GSCs.

Phenotype	Cell Lines	*TRPA1* mRNA		*TRPV1* mRNA	
		Relative mRNA Expression ± SD	Fold Expression	Relative mRNA Expression ± SD	Fold Expression
	NSC	0.151 ± 0.014	1.00	0.554 ± 0.023	1.00
PN-like	GSC#1	0.961 ± 0.031	6.36	0.314 ± 0.089	0.57
MES-like	GSC#83	8.881 ± 0.101	58.81	1.774 ± 0.051	3.20
PN-like	GSC#23C	0.071 ± 0.003	0.47	0.163 ± 0.004	0.29
PN-like	GSC#28	0.292 ± 0.043	1.93	0.217 ± 0.023	0.39
MES-like	GSC#30	0.302 ± 0.003	2.00	0.015 ± 0.003	0.03
PN-like	GSC#68	0.452 ± 0.013	2.99	0.543 ± 0.005	0.98
PN-like	GSC#70	0.881 ± 0.054	5.83	0.692 ± 0.022	1.25

mRNA samples extracted from NSCs and GSCs. Data represents relative mRNA levels and fold expression of individual gene in GSCs compared to the NSCs. The expression levels were normalized to the average Ct value of two housekeeping genes (*GAPDH* and *RPLP0*) and calculated by the2^−ΔΔCt^ method.

**Table 3:** *Aml1b* and *Aml1c* splice variants mRNA expression in D-GSCs.

Phenotype	Cell Lines	*Aml1b* mRNA		*Aml1c* mRNA	
		Relative mRNA Expression ± SD	Fold Expression	Relative mRNA Expression ± SD	Fold Expression
PN-like	D-GSC#1	2.621 ± 0.031	2.15	2.813 ± 0.089	2.26
MES-like	D-GSC#83	5.056 ± 2.101	2.08	10.085 ± 0.651	2.18
PN-like	D-GSC#23C	1.722 ± 0.032	2.39	1.988 ± 0.021	2.38
PN-like	D-GSC#28	2.776 ± 0.035	3.79	3.500 ± 0.023	3.23
MES-like	D-GSC#30	7.810 ± 0.013	4.00	0.646 ± 0.004	1.99
PN-like	D-GSC#68	2.071 ± 0.107	1.20	0.612 ± 0.005	2.85
PN-like	D-GSC#70	1.303 ± 0.046	1.06	0.998 ± 0.013	3.08

mRNA samples extracted from GSCs and D-GSCs at 14 days of differentiation. Data represents relative mRNA levels and fold expression of individual gene in D-GSCs compared to the respective GSCs (=1). The expression levels were normalized to the average Ct value of two housekeeping genes (*GAPDH* and *RPLP0*) and calculated by the 2^−ΔΔCt^ method.

**Table 4:** *TRPA1* and *TRPV1* channels mRNA expression in D-GSCs.

Phenotype	Cell Lines	*TRPA1* mRNA		*TRPV1* mRNA	
		Relative mRNA Expression ± SD	Fold Expression	Relative mRNA Expression ± SD	Fold Expression
PN-like	D-GSC#1	4.622 ± 0.031	4.81	0.187 ± 0.089	0.34
MES-like	D-GSC#83	38.851 ± 0.101	4.49	1.202 ± 0.051	0.68
PN-like	D-GSC#23C	4.994 ± 0.221	212.93	0.113 ± 0.011	0.69
PN-like	D-GSC#28	0.122 ± 0.001	0.42	0.131 ± 0.001	0.60
MES-like	D-GSC#30	1.143 ± 0.013	3.78	0.007 ± 0.003	0.47
PN-like	D-GSC#68	0.292 ± 0.022	0.65	0.363 ± 0.023	0.67
PN-like	D-GSC#70	0.227 ± 0.011	0.26	0.233 ± 0.014	0.34

mRNA samples extracted from GSCs and D-GSCs at 14 days of differentiation. Data represents relative mRNA levels and fold expression of individual genes in D-GSCs compared to the respective GSCs. The expression levels were normalized to the average Ct value of two housekeeping genes (*GAPDH* and *RPLP0*) and calculated by the 2^−ΔΔCt^method.

**Table 5:** Analysis of stem cell signaling pathway in PN-like GSC#1 during differentiation.

Upregulated Genes	Fold Expression ± SD	Downregulated Genes	Fold Expression ± SD
*ACVR1*	2.54 ± 0.20	*E2F5*	−2.45 ± 0.12
*AMHR2*	3.81 ± 0.10	*FZD2*	−2.86 ± 0.11
*BMPR2*	2.66 ± 0.32	*LIFR*	−2.91 ± 0.01
*CDX2*	2.40 ± 0.15	*NOTCH3*	−2.95 ± 0.15
*FZD1*	2.77 ± 0.03	*NOTCH4*	−2.00 ± 0.29
*FZD3*	2.69 ± 0.10	*SMAD3*	−2.15 ± 0.08
*SMAD2*	2.67 ± 0.10	*STAT3*	−5.06 ± 0.50

Stem Cell Signaling Pathway Finder PCR array in mRNA samples extracted from GSC#1 and D-GSC#1 after 7 days of culture. Values represent fold differences of individual gene expression in D-GSC#1 compared to GSC#1. The expression levels were normalized to the average Ct value of two housekeeping genes (*GAPDH* and *RPLP0*) and calculated by the ΔΔCt method. Genes, whose expression is, at least, 2.00-fold up/downregulated, are shown.

**Table 6:** Analysis of stem cell signaling pathway in MES-like GSC#83 during differentiation.

Upregulated Genes	Fold Expression ± SD	Downregulated Genes	Fold Expression ± SD
*ACVR1*	2.85 ± 0.10	*FGFR4*	−11.64 ± 0.08
*AMHR2*	2.55 ± 0.30	*FZD1*	−10.26 ± 0.01
*BMPR1B*	2.90 ± 0.29	*LRP6*	−4.83 ± 0.11
*CDX2*	2.00 ± 0.05	*LTBP2*	−7.75 ± 0.10
*FGFR2*	6.70 ± 0.30	*NFAT5*	−2.24 ± 0.09
*FZD9*	3.00 ± 0.15	*NOTCH4*	−8.84 ± 0.51
*IL6ST*	3.34 ± 0.10	*RGMA*	−2.64 ± 0.61
*LIFR*	2.40 ± 0.12	*SMAD3*	−3.33 ± 0.31
*RBPJL*	2.05 ± 0.13	*TGFBR2*	−6.40 ± 0.72
*SMAD9*	4.70 ± 0.10		

Stem Cell Signaling Pathway Finder PCR array in mRNA samples extracted from GSC#83 and D-GSC#83 after 7 days of culture. Values represent fold differences of individual gene expression in D-GSC#83 compared to GSC#83. The expression levels were normalized to the average Ct value of two housekeeping genes (*GAPDH* and *RPLP0*) and calculated by the 2^−ΔΔCt^ method. Genes, whose expression was, at least, 2.00-fold up/downregulated, are shown.

## Data Availability

The data presented in this study are available on request from the corresponding author.

## Supplementary Materials

The following are available online at https://www.mdpi.com/article/10.3390/cells10082052/s1, Figure S1: Phenotype characterization of GSCs, Table S1: Aml1b and Aml1c splice variants mRNA fold expression in D-GSCs expressed as relative fold with respect to D-NSC, Table S2: TRPA1 and TRPV1channels mRNA fold expression in D-GSCs expressed as relative fold with respect to D-NSC.
