# Supplementary material for: ERK Phosphorylation Regulates the Aml1/Runx1 Splice Variants and the TRP Channels Expression during the Differentiation of Glioma Stem Cell Lines
Source: Cells. 2021 Aug 10;10(8):2052. doi: 10.3390/cells10082052 (PMC8391729; doi:10.3390/cells10082052)
Supplement: Supplementary file 1 [file cells-10-02052-s001.zip › cells-1328981-supplementary.pdf]

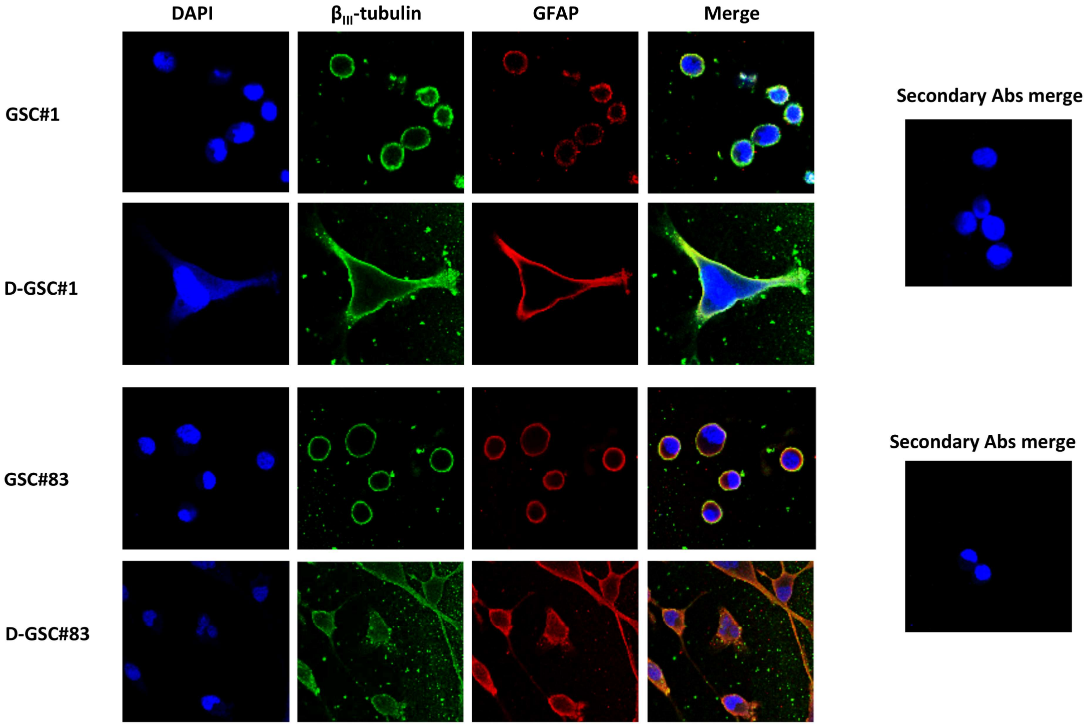

Figure S1. Changes in the expression of neural markers during differentiation of different GSC lines: GSCs and D-GSCs were fixed, permeabilized, and stained with anti-human betaIII-tubulin and GFAP Abs followed by Alexa Fluor-488 and Alexa Fluor-594 secondary Abs, respectively. 40,6-diamidino-2-phenylindole (DAPI) was used to counterstain nuclei. Magnification 60x.

Table S1. Aml1b and Aml1c splice variants mRNA fold expression in D-GSCs expressed as relative fold with respect to D-NSC.

Phenotype	Cell lines	Aml1b mRNA Fold expression	Aml1c mRNA Fold expression
	D-NSC	1.00	1.00
PN-like	D-GSC#1	2.61	2.71
MES-like	D-GSC#83	5.04	12.53
PN-like	D-GSC#23C	1.77	2.46
PN-like	D-GSC#28	2.77	1.15
MES-like	D-GSC#30	7.79	0.80
PN-like	D-GSC#68	2.06	0.76
PN-like	D-GSC#70	1.06	0.40

mRNA samples extracted from D-NSCs and D-GSCs. Fold expression represents fold difference of individual gene expression in D-GSCs compared to the D-NSCs. The expression levels were normalized to the average Ct value of two housekeeping genes (GAPDH and RPLP0) and calculated by the $\Delta\Delta$Ct method.

Table S2. TRPA1 and TRPV1 channels mRNA fold expression in D-GSCs expressed as relative fold with respect to D-NSC.

**Phenotype**	**Cell lines**	**TRPA1 mRNA**	**TRPV1 mRNA**
		**Fold expression**	**Fold expression**
	D-NSC	1.00	1.00
PN-like	D-GSC#1	4.31	0.15
MES-like	D-GSC#83	37.21	0.31
PN-like	D-GSC#23C	4.67	0.13
PN-like	D-GSC#28	0.11	0.11
MES-like	D-GSC#30	1.07	0.00
PN-like	D-GSC#68	0.27	0.30
PN-like	D-GSC#70	0.21	0.19

mRNA samples extracted from D-NSCs and D-GSCs. Fold expression represents fold difference of individual gene expression in D-GSCs compared to the D-NSCs. The expression levels were normalized to the average Ct value of two housekeeping genes (GAPDH and RPLP0) and calculated by the ΔΔCt method.
